# Magnesium in hypertension: mechanisms and clinical implications

**DOI:** 10.3389/fphys.2024.1363975

**Published:** 2024-04-10

**Authors:** Zain AlShanableh, Evan C. Ray

**Affiliations:** Renal-Electrolyte Division, UPMC and University of Pittsburgh School of Medicine, Pittsburgh, PA, United States

**Keywords:** magnesium, hypertension, aldosterone, NLPR3 inflammasome, isolevuglandins (IsoLG)

## Abstract

Hypertension is associated with increased risk of cardiovascular disease and death. Evidence suggests that Mg^2+^ depletion contributes to hypertension. It is estimated that 25% or more of the United States population experiences chronic, latent Mg^2+^ depletion. This review explores mechanisms by which Mg^2+^ influences blood pressure, modifying risk of hypertension and complicating its treatment. Mechanisms addressed include effects upon i) sympathetic tone, via the modulation of N-methyl-D-aspartate (NMDA) receptor and N-type Ca^2+^ channel activity, influencing catecholamine release from sympathetic nerve endings; ii) vascular tone, via alteration of L-type Ca^2+^ and endothelial nitric oxide synthase (eNOS) activity and prostacyclin release; iii) renal K^+^ handling, influencing systemic K^+^ balance and potentially indirectly influencing blood pressure; iv) aldosterone secretion from the adrenal cortex; and v) modulation of pro-hypertensive inflammatory processes in dendritic cells and macrophages, including activation of the NLR family pyrin domain containing 3 (NLRP3) inflammasome and stimulation of isolevuglandin (IsoLG) production. Discovery of these mechanisms has furthered our understanding of the pathogenesis of hypertension, with implications for treatment and has highlighted the role of Mg^2+^ balance in hypertension and cardiovascular disease.

## Introduction

Magnesium is an essential ion and is required for normal health, including the cardiovascular system (de Baaij et al., 2015). Numerous studies have explored the association between Mg^2+^ and blood pressure, and evidence suggests that Mg^2+^ depletion contributes to hypertension. This review will focus on the effect of Mg^2+^ on blood pressure and hypertension and will discuss different mechanisms by which Mg^2+^ influences blood pressure.

Hypertension is widely prevalent. In the United States, it affects 119.9 million adults - nearly half the population (United States Centers for Disease Control and Prevention, 2015). Hypertension increases the risk of cardiovascular disease and stroke (Fuchs and Whelton, 2020). In 2020, it was estimated that cardiovascular disease contributed to around 19,000,000 deaths globally (Tsao et al., 2022).

Systemic Mg^2+^ depletion is common. Reports estimate that 25% or more of the population of the United States experiences chronic, latent Mg^2+^ depletion (Lowenstein and Stanton, 1986; Rosanoff et al., 2022). Mg^2+^ depletion is under-appreciated clinically, partly because reference ranges for plasma Mg^2+^ in clinical laboratories are based upon population distribution rather than healthy levels. Study groups in the United States (Costello et al., 2016) and in Germany (Micke et al., 2021), have independently recommended an evidence-based lower limit of normal for serum Mg^2+^ of 2.07 mg/dL (0.85 mmol/L). However, a 2022 study found that in 41 out of 43 medical centers in 16 countries employ a lower limit beneath this recommended threshold (Rosanoff et al., 2022). Prevalence of Mg^2+^ depletion appears even higher in individuals with hypertension, as intracellular Mg^2+^ levels are lower in hypertensive individuals than control individuals (Resnick et al., 1984; Touyz et al., 1992). Plasma Mg^2+^, a less sensitive indicator of Mg^2+^ deficiency, was found to be lower in hypertensive individuals with elevated renin but not in other hypertensive individuals (Resnick et al., 1983).

Dietary Mg^2+^ insufficiency is a common contributor to systemic Mg^2+^ depletion. The United States estimated average requirement (EAR) for Mg^2+^ is 255 mg/day for women aged 19–30 years, increasing to 265 mg/day for women aged ≥31 years. For men aged 19–30 the EAR is 330 mg/day, increasing to 350 mg/day for men aged ≥31 years (Rosanoff et al., 2012). According to the United States National Health and Nutrition Examination Survey (NHANES) 2013–2016 report, this EAR was not met in nearly half (48%) of the U.S. population (USDA Agricultural Research Service, 2019).

Commonly prescribed medications also contribute to systemic Mg^2+^ depletion (Ray et al., 2023). Given the contribution of Mg^2+^ depletion to cardiovascular disease (Kolte et al., 2014); it is particularly concerning that treatment with a first-line therapy for hypertension, thiazide-type diuretics, promotes urinary Mg^2+^ wasting and systemic Mg^2+^ depletion (Hollifield, 1986).

## Evidence for a relationship between Mg^2+^ and hypertension

The earliest findings of an effect of Mg^2+^ upon blood pressure were reported more than 100 years ago, when Kenneth Blackfan (subsequently famous for his description of Diamond Blackfan anemia) and Charles McKhann described “a rapid fall in blood pressure” in children with glomerulonephritis and severely elevated blood pressure (Blackfan and Mills, 1923; Blackfan and McKhann, 1931). Studies much later would seek to understand the circumstances under which Mg^2+^ can attenuate hypertension.

Several studies have explored the relationship between dietary Mg^2+^ and blood pressure in experimental animals, with mixed results. In rats, some studies show increased blood pressure with dietary Mg^2+^ restriction (Berthelot and Esposito, 1983; Altura et al., 1984; Laurant et al., 1999; Murasato et al., 1999; Carlin Schooley and Franz, 2002; Blache et al., 2006), others do not (Itokawa et al., 1974; Overlack et al., 1987; Lowney et al., 1988; Luthringer et al., 1988; Evans et al., 1989; Liu et al., 1994; Laurant et al., 1997; Tomiyasu et al., 1998). No doubt these discrepancies reflect differences in experimental details such as strains used, severity of dietary Mg^2+^ restriction, and duration. In mice, dietary Mg^2+^ deficiency was shown to stimulate salt-sensitive increase in blood pressure in DBA but not C57Bl/6J mice (Kumagai et al., 2021). However, in C56Bl/6J mice, a Mg^2+^-restricted diet did increase blood pressure raising-effects of sympathetic stimulation. In mice of the sv129 background, dietary Mg^2+^ restriction increased blood pressure by 21 days until sacrifice at 5 weeks (Pitzer Mutchler et al., 2023). Intravenous Mg^2+^ infusion in rats attenuates increases in blood pressure resulting from angiotensin II (Atarashi et al., 1990) or sympathetic nerve stimulation (Shimosawa et al., 2004).

Observational studies have explored the correlation between circulating Mg^2+^ in humans and blood pressure. A structured review and subgroup analysis of observational studies explored the association between dietary Mg^2+^ intake and blood pressure. Findings suggested an inverse relationship between dietary Mg^2+^ intake and blood pressure, though heterogeneity in study methods complicated interpretation (Mizushima et al., 1998). In an observational study of 1,000 ambulatory hypertensive patients, hypomagnesemia was associated with worsened hypertension, as indicated by a greater number of prescribed anti-hypertensive medications (Whang et al., 1982). Plasma Mg^2+^ levels are lower in individuals with untreated elevated systolic blood pressure and diastolic blood pressure than in normotensive controls (Rodríguez-Moran and Guerrero-Romero, 2014; Rodríguez-Ramírez et al., 2015).

Numerous human clinical trials have examined the effects of Mg^2+^ supplementation in management of hypertension. A meta-analysis by Zhang et al., pooled 24 randomized controlled trials (RCTs) with a total of 2,028 participants. They concluded that supplementation of Mg^2+^ at a mean dose of 368 mg/day for a median period of 3 months resulted in 2 mmHg reduction in SBP (95% CI, 0.43–3.58 mmHg; *p* = 0.01) and 1.78 mmHg reduction in DBP (CI, 0.73–2.82 mmHg; *p* = 0.001) (Zhang et al., 2016). Another meta-analysis by Dibaba et al., reviewed 11 RCTs, including 543 participants. Mg^2+^ supplementation of 365–450 mg/day for a mean of 3.6 months significantly reduced SBP by a mean of 4.18 mmHg (standard mean difference: −0.20; 95% CI: −0.37, −0.03) and DBP by a mean of 2.27 mmHg (standard mean difference: −0.27; 95% CI: −0.52, −0.03) (Dibaba et al., 2017). Rosanoff et al., conducted a meta-analysis of 49 clinical trials that stratified study participants into the following groups: 1) untreated hypertensives 2) uncontrolled hypertensives 3) controlled hypertensives 4) normotensive subjects. They found that a Mg^2+^ dose of ≥240 mg/day decreases BP in treated but uncontrolled hypertensive individuals, and a dose of >600 mg/day lowers BP in untreated hypertensives. There was no change in BP in individuals who were normotensive, had controlled HTN, or were Mg^2+^-replete (Rosanoff et al., 2021). A meta-analysis of seven RCTs examining hypertensive individuals with diabetes found that Mg^2+^ supplementation reduced systolic blood pressure by 5.78 and diastolic blood pressure by 2.5 mmHg (Asbaghi et al., 2021). This is of particular interest, as diabetic patients tend to be Mg^2+^ depleted (Ray et al., 2023).

Together, these findings suggest that systemic Mg^2+^ depletion promotes increased blood pressure in patients with hypertension.

## Mechanisms influencing blood pressure

**FIGURE 1:**
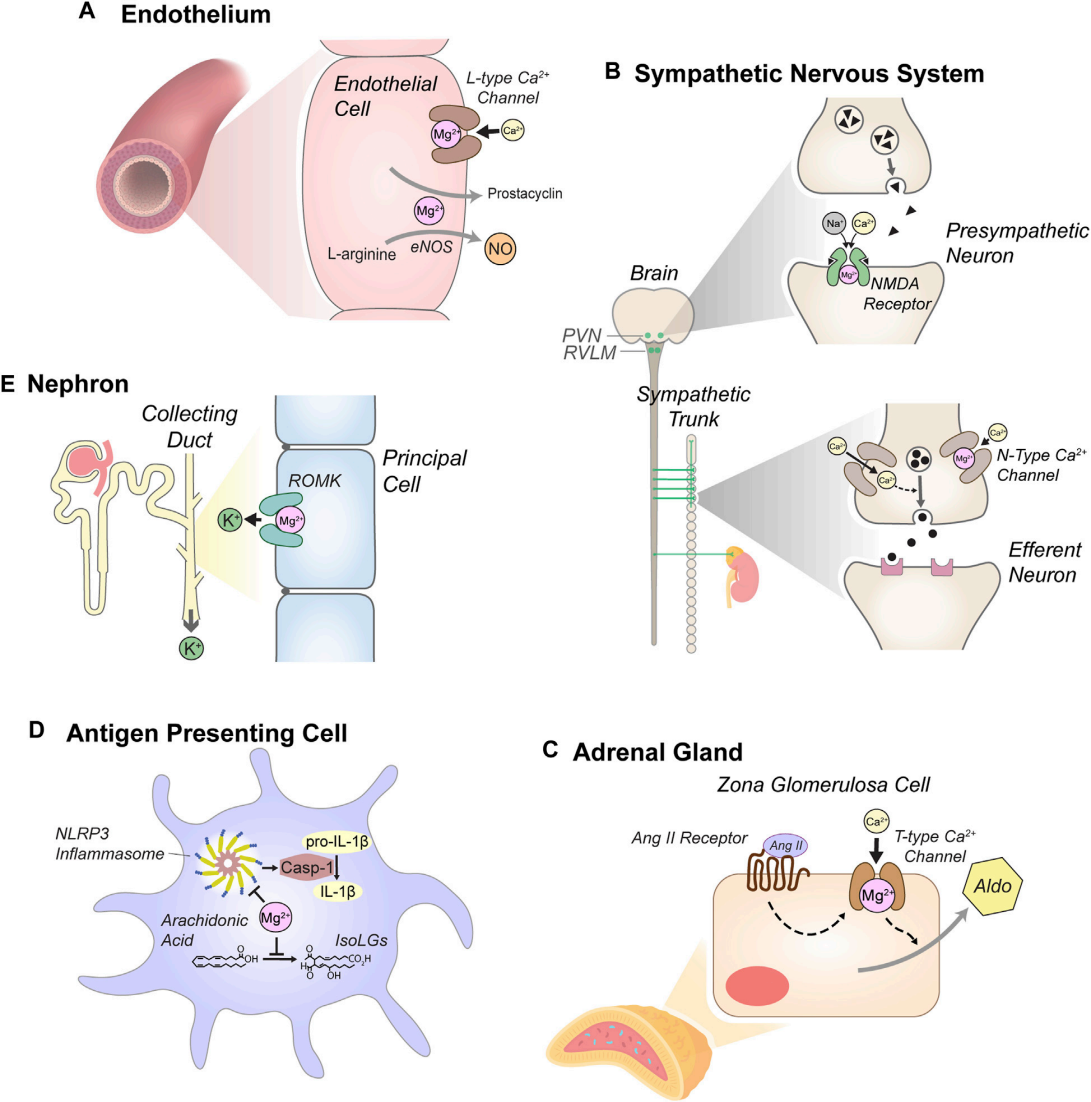
Anti-hypertensive effects of Mg^2+^. **(A)** Mg^2+^ reduces vascular tone through actions in endothelial cells, including blockade of *L-type* Ca^2+^ channels and by supporting secretion of prostacyclin and nitric oxide (NO). *L-type* Ca^2+^ channel blockade and other mechanisms also reduce intracellular Ca^2+^ in myocytes, attenuating cell contraction (not depicted). **(B)** Mg^2+^ attenuates sympathetic tone. Blockade of *N-methyl-D-aspartate* (NMDA) receptors in the *paraventricular nucleus* (PVN) of the hypothalamus and the *rostral ventrolateral medulla* (RVLM) attenuates pre-sympathetic neuron activity. In sympathetic ganglia, Mg^2+^ reduces *N-type* Ca^2+^ channel activity, modulating stimulation of efferent sympathetic neurons. **(C)** Mg^2+^ reduces aldosterone secretion in the adrenal cortex. Mg^2+^ blockade of *T-type* Ca^2+^ channels in *zona glomerulosa* cells modulates stimulation of aldosterone (Aldo) secretion by angiotensin II (Ang II). **(D)** Mg^2+^-depletion stimulates antigen presenting cells (dendritic cells and monocytes). Mg^2+^ depletion enhances expression of NLR family pyrin domain containing 3 (NLRP3), a key component of the inflammasome, which activates caspase-1 (Casp-1). Casp-1 stimulates production of pro-inflammatory cytokines, including IL-1β. Mg^2+^ depletion also enhances production of isolevuglandins (IsoLGs), reactive aldehydes that promote pro-hypertensive inflammation. **(E)** Mg^2+^ reduces urinary K^+^ excretion through blockade of the renal outer medullary K^+^ channel (ROMK) in the distal nephron, attenuating systemic K^+^-depletion. Although K^+^-depletion can stimulate the thiazide-sensitive NaCl cotransporter (NCC), this mechanism does not appear to contribute to increased blood pressure in the context of systemic Mg^2+^ depletion (see text).

Because of its vast physiologic effects, Mg^2+^ depletion likely influences blood pressure via multiple mechanisms, as discussed below and summarized in Figure 1.

### Vascular tone

Effects of Mg^2+^ on vascular tone may contribute to its influence upon blood pressure. Empiric evidence for an effect of Mg^2+^ upon vascular tone is demonstrated by the observations that decreased extracellular Mg^2+^ or systemic Mg^2+^ depletion in animals produces vasospasm and reduces microvascular blood flow (Altura and Turlapaty, 1982; Altura et al., 1983; Altura et al., 1984). Humans given an acute MgSO_4_ infusion exhibit increased renal blood flow, despite reduced blood pressure (Nadler et al., 1987).

Vascular smooth muscle constriction is stimulated when Ca^2+^ enters smooth muscle cytosol via L-type voltage-gated Ca^2+^ channels. Intracellular Ca^2+^ stimulates phospholipase C and production of diacylglycerol (DG) and inositol 1,4,5-trisphosphate (IP_3_). IP_3_ activates the IP_3_ receptor, releasing Ca^2+^ from the sarcoplasmic reticulum. Cytosolic Ca^2+^ then binds to calmodulin, activating myosin light chain kinase (MLCK). Activated MLCK phosphorylates the myosin light chain, stimulating interaction of actin and myosin, and eliciting cell contraction (Webb, 2003).

Intracellular Mg^2+^ attenuates myocyte contraction via several mechanisms. Mg^2+^ diminishes cellular Ca^2+^ entry via L-type voltage gated Ca^2+^ channels (Zhang et al., 2007; Sharma et al., 2012). Intracellular Mg^2+^ inhibits Ca^2+^-stimulated Ca^2+^-release from the sarcoplasmic reticulum, at least in cardiac muscle (Dunnett and Nayler, 1978). The sarcoplasmic reticulum Ca^2+^-ATPase, which is required to return released Ca^2+^ to intracellular stores, requires Mg^2+^ for activity (Hasselbach et al., 1981). Consequently, low Mg^2+^ conditions prolong elevation of intracellular Ca^2+^ following release from intracellular stores (Gasallaherraiz et al., 1995). Thus, Mg^2+^ reduces smooth muscle contraction.

*Prostacyclin release:* Effects of systemic Mg^2+^ status upon vascular tone may be mediated, in part, by effects on prostacyclin release. Prostacyclin (PGI_2_) is recognized to have important systemic vasodilatory effects (Zhao and Richardson, 1990). In the kidney, prostacyclin is critical for maintaining vasodilation and blood flow in the context of extrarenal vasoconstriction. Mice lacking prostacyclin synthase exhibit hypertension, thickening of the aortic medial and adventitial layers, and nephrosclerosis (Yokoyama et al., 2002). In humans, a repeat polymorphism in the promoter region of the prostacyclin synthase gene was found to reduce prostacyclin synthase transcription and to be associated with increased odds of hypertension (Iwai et al., 1999).

Mg^2+^ modulates vascular prostacyclin release. In cultured vascular endothelial cells or smooth muscle cells, increased extracellular Mg^2+^ stimulated prostacyclin secretion (Briel et al., 1987; Satake et al., 2004). In the rat deoxycorticosterone acetate (DOCA)-salt model of hypertension, a Mg^2+^-enriched diet significantly increased PGI_2_ levels (Laurant et al., 1992). Infusion of MgSO_4_ into humans enhanced urinary excretion of immunoreactive *6-ketoprostaglandin* F1α (6-keto-PGF_1a_), a stable break-down product of PGI_2_, while reducing blood pressure (Nadler et al., 1987). The importance of prostaglandin synthesis in this blood pressure effect was demonstrated by the observation that cyclooxygenase inhibition prevented the decrease in blood pressure and increase in renal blood flow. Moreover, the Mg^2+^-stimulated increase in PGI_2_ release was blocked by the calcium channel blocker, nifedipine, suggesting that the influence of Mg^2+^ on cyclooxygenase is dependent upon Ca^2+^ entry into cells.

*Nitric oxide metabolism:* Mg^2+^ also influences vascular tone through effects on nitric oxide (NO). Nitric oxide is an endogenous vasodilator produced in endothelial cells from L-arginine by endothelial NO synthase (eNOS) (Rees et al., 1989; Gamboa et al., 2007). In cultured endothelial cells, NO production was roughly 3-fold higher in cells grown in high (5 mM, or 12 mg/dL) than in control (1 mM, or 2.4 mg/dL) extracellular Mg^2+^ (Maier et al., 2004). This finding was attributed to an observed increase in eNOS protein abundance in cells grown in high Mg^2+^.

Altered NO release may contribute to effects of Mg^2+^ upon NO signaling. In mouse aorta and mesenteric vessels, endothelium-dependent, Mg^2+^-induced arterial relaxation is attenuated by blockade of eNOS activity with N (gamma)-nitro-L-arginine methyl ester (L-NAME) (Kudryavtseva et al., 2024). In canine coronary arteries, Mg^2+^-free conditions attenuated acetylcholine and ADP-stimulated, NO-dependent reduction in arterial tone (Pearson et al., 1998). The Ca^2+^ ionophore A23187, which induces endothelial NO release independently of receptor-mediated signaling mechanisms, reduced arterial tension in a Mg^2+^-independent fashion. Bradykinin-stimulated vascular relaxation, which occurs via endothelium-dependent, but NO-independent mechanisms, was unaffected (Pearson et al., 1998). These findings suggest that Mg^2+^ is required for stimulation of NO release but not for NO-stimulated relaxation. Evidence that Mg^2+^ influences NO signaling in humans comes from a study examining flow-mediated vasodilation of the brachial artery (FMD), a process that is at least partly NO mediated (Green et al., 2014). Oral Mg^2+^ supplementation in individuals with coronary artery disease significantly improved FMD and exercise tolerance (Shechter et al., 2000).

Mg^2+^ likely also promotes relaxation via additional, NO-independent pathways. Blockade of SK and IK Ca^2+^-activated K^+^ channels, which participate in endothelium-derived relaxation factor-stimulated arterial relaxation, blunted Mg^2+^-dependent arterial relaxation additively with eNOS inhibition (Kudryavtseva et al., 2024).

Together, these observations suggest that systemic Mg^2+^ status could influence blood pressure through multiple effects on vascular tone.

### Sympathetic tone

Mg^2+^ exerts an inhibitory effect on the sympathetic nervous system, whereas Mg^2+^ deficiency stimulates sympathetic tone.

Effects of Mg^2+^ upon the sympathetic nervous system are mediated, in part, through modulation of N-methyl-D-aspartate (NMDA) receptor activity. The NMDA receptor is a Ca^2+^-selective ion channel that opens in response to NMDA and L-Glutamate (L-Glu) (Dingledine et al., 1999; Kagiyama et al., 2001). NMDA receptor activity in the rostral ventrolateral medulla (RVLM) and hypothalamic paraventricular nucleus (PVN) increase sympathetic outflow and blood pressure (Dampney et al., 2003; Li and Pan, 2017). NMDA receptor activity is negatively regulated by Mg^2+^ (Dingledine et al., 1999). Thus, attenuation of NMDA receptor activity by Mg^2+^ may be expected to reduce blood pressure.

In support of this hypothesis, Kagiyama et al., studied the effect of Mg^2+^ in the RVLM upon blood pressure. Injection of magnesium sulfate (MgSO_4_) into the RVLM exerted a dose-dependent attenuation of increased blood pressure occurring in response to NMDA injection (Kagiyama et al., 2001).

Mg^2+^ also negatively influences sympathetic tone through modulation of catecholamine release from peripheral nerve endings and by blocking N-type Ca^2+^ channels at nerve endings (Shimosawa et al., 2004). In neuronally differentiated PC12 cells, N-type Ca^2+^ channel activity was decreased by elevated extracellular Mg^2+^ and increased by reduced extracellular Mg^2+^. With cytosolic Ca^2+^ being a major stimulus for catecholamine release, low extracellular Mg^2+^ buffer enhanced norepinephrine release from the periarterial plexus of the mesenteric artery compared to control or high Mg^2+^ buffer. Urinary catecholamine excretion was found to be more than two-fold higher in Mg^2+^ deficient rats than control rats (Murasato et al., 1999). Mg^2+^ infusion also attenuated sympathetically mediated reflex tachycardia following hydralazine infusion (Shimosawa et al., 2004). In addition to reducing norepinephrine release from sympathetic neurons, Mg^2+^ also increased norepinephrine uptake in isolated adrenergic nerve granules, suggesting an influence upon norepinephrine reuptake in the synaptic cleft (von Euler and Lishajko, 1963; von Euler and Lishajko, 1973).

Effects of Mg^2+^ on the sympathetic nervous system have also been demonstrated in human subjects. James et al., studied the effect of MgSO_4_ infusion upon simulation of catecholamine release and blood pressure in response to endotracheal intubation. In controls, intubation rapidly increased circulating epinephrine levels, norepinephrine levels, and systolic blood pressure. MgSO_4_ infusion attenuated the increase in each of these (James et al., 1989).

Thus, Mg^2+^ modulates peripheral sympathetic nervous system activity, reducing blood pressure.

### Effects on K^+^ and Na^+^ handling

Systemic Mg^2+^ status could influence blood pressure indirectly, through effects on handling of K^+^ and Na^+^.

Bodily K^+^ balance influences blood pressure. Several meta-analyses of clinical trials find that K^+^ supplementation reduces blood pressure in hypertensive individuals (van Bommel and Cleophas, 2012; Poorolajal et al., 2017; Filippini et al., 2020). The influence of K^+^ upon blood pressure is likely mediated by multiple mechanisms, including effects upon vascular tone and upon extracellular fluid volume. In Dahl salt-sensitive rats, a high K^+^-diet promotes vascular relaxation (Raij et al., 1988). Fluid volume effects may occur secondary to enhanced tubular Na^+^ reabsorption in the context of systemic K^+^ depletion. In the kidney’s proximal convoluted tubule (PCT), a low K^+^ diet enhanced protein abundance of the Na^+^/H^+^ exchanger, type 3 (NHE3), promoting Na^+^/H^+^ exchange (Shirley et al., 1990; Soleimani et al., 1990; Elkjær et al., 2002). In the distal convoluted tubule (DCT), K^+^ deficiency stimulates phosphorylation-mediated activation of sodium-chloride cotransporter (NCC) through modulation of the WNK/SPAK/OSR1 (with no lysine/SPS1-related proline-alanine-rich kinase/oxidative stress-responsive kinase 1) signal transduction pathway (Terker et al., 2015).

Mg^2+^ depletion promotes K^+^ depletion. Because serum K^+^ represents only 2% of total body K^+^, even when plasma or serum K^+^ is not appreciably reduced, intracellular and total body K^+^ stores can be depleted (Patrick, 1977; Brown, 1984). In rats subjected to dietary Mg^2+^ restriction, intramuscular K^+^ declined (Macintyre and Davidsson, 1958; Manitius and Epstein, 1963; Whang and Welt, 1963; Ginn et al., 1967; Dørup and Clausen, 1993). This was associated with decreased whole body K^+^ following prolonged (60-day) dietary Mg^2+^ restriction (Whang and Welt, 1963). In human subjects given a low Mg^2+^ diet, urinary K^+^ excretion increased overall and total exchangeable K^+^ decreased (Shils, 1969). Intracellular Mg^2+^ depletion is thought to promote urinary K^+^ excretion through loss of voltage-dependent blockade of the outer medullary K^+^ channel (ROMK) in the kidney tubule, enhancing tubular K^+^ secretion (Huang and Kuo, 2007). Additionally, systemic Mg^2+^ depletion increases circulating aldosterone levels (discussed below), enhancing urinary K^+^ excretion in exchange for Na^+^ reabsorption.

Taken together, these findings suggest the hypothesis that Mg^2+^ depletion could contribute to urinary Na^+^ retention and increased blood pressure by stimulating NCC activity in the DCT. Surprisingly, rats given a low Mg^2+^ diet exhibit reduced NCC expression (Fanestil et al., 1999). Ferdaus et al. confirmed these findings in mice and found that dietary Mg^2+^ depletion reduced both total and phosphorylated NCC protein abundance in the kidney. NCC mRNA levels were unchanged, suggesting post-transcriptional effects on NCC expression. The hypothesis that dietary Mg^2+^ restriction may stimulate NCC degradation was supported by the observation that kidney-specific deletion of the ubiquitin ligase NEDD4-2 blocked downregulation of NCC by dietary Mg^2+^ restriction. Dietary Mg^2+^ depletion even blocked the ability of a K^+^-restricted diet to increase total and phosphorylated-NCC protein abundance, providing further evidence that the influence of Mg^2+^ depletion on blood pressure is not NCC-mediated.

Systemic K^+^ depletion may influence Na^+^ handling in other portions of the nephron, such as the thick ascending loop of Henle. The Na-K-Cl co-transporter (NKCC2) in the thick ascending limb (TAL) is also modulated by intracellular WNK/SPAK/OSR1 pathway (Moriguchi et al., 2005; Rinehart et al., 2005; Liu et al., 2011; Richardson et al., 2011; Park et al., 2013; Terker et al., 2018; Marcoux et al., 2019). Given the ability of systemic K^+^ status to influence the WNK/SPAK/OSR1 pathway, it seems likely that differences in Mg^2+^ homeostasis may influence this pathway via changes in systemic K^+^, but we are unaware of data directly exploring this hypothesis.

Mg^2+^ could also influence Na^+^ reabsorption in the TAL through modulation of the calcium-sensing receptor (CaSR). Activation of the CaSR on the basolateral surface of cortical TAL cells reduces apical K^+^ channel activity (Wang et al., 1996). Impaired cellular K^+^ efflux impairs Na^+^ and Cl^−^ reabsorption through NKCC2, producing a loop diuretic-like effect. Mg^2+^, like Ca^2+^, can activate the CaSR, which may explain the earlier observation that intravenous Mg^2+^ infusion can decrease TAL Na^+^ reabsorption (Ploth et al., 1976). Whether changes in plasma Mg^2+^ within the physiologic range modulate CaSR activity and TAL NaCl reabsorption remains unclear.

### Renin-angiotensin-aldosterone system

Systemic Mg^2+^ status may also influence Na^+^ and K^+^ handling via modulation of the renin-angiotensin-aldosterone system. In laboratory animals, dietary Mg^2+^ restriction appears to stimulate aldosterone levels. Sapna et al. found that in rats, 6 days on low Mg^2+^ chow resulted in serum aldosterone of 205.0 ± 66.2 pg/mL, which was not significantly higher than 138.1 ± 80.8 pg/mL seen on control chow (Sapna et al., 2006). However. Laurant et al. observed an increase in plasma aldosterone in rats given Mg^2+^-deficient diet for two and 21 weeks (Laurant et al., 1999). Stimulation of aldosterone levels by dietary Mg^2+^ restriction seems to occur independently of extracellular fluid volume status, as dietary Mg^2+^ depletion continued to stimulate increased serum aldosterone even in animals given a high Na^+^ diet (Solounias and Schwartz, 1975).

Acute intravenous Mg^2+^ administration also reduces aldosterone levels. A study examining six “healthy,” normotensive volunteers infused MgSO_4_ at a rate of 0.6 mg/h (5 mEq/hr) and found that plasma aldosterone levels decreased to 4 ± 0.8 ng/dL (111 ± 22 pmol/L) compared with 6 ± 0.2 ng/dL (166 ± 5.5 pmol/L) in controls (*p* < 0.05) (Ichihara et al., 1993). This occurred despite an increase in plasma renin activity. Corica et al. infused 3 gm (24 mEq) of MgSO_4_ into “healthy” volunteers and observed reduced serum aldosterone from 18.97 ± 11 ng/dL (526 ± 305 pmol/L) to 6.34 ± 5 ng/dL (176 ± 139 pmol/L) (Corica et al., 1996). This effect did not appear to be fluid volume mediated, as atrial natriuretic peptide levels did not change, and a control infusion with isotonic saline had no significant impact on aldosterone levels. Thus, Mg^2+^ sulfate infusion reduces aldosterone levels in humans, at least acutely.

Despite these observations, oral supplementation studies in humans have largely failed to demonstrate reduction in circulating aldosterone. One study gave 365 mg (15 mmol) of Mg^2+^, as Mg^2+^ aspartate, to 17 subjects for 4 weeks (Cappuccio et al., 1985). Neither blood pressure nor aldosterone changed compared with participants receiving placebo. Participants had a mean baseline serum Mg^2+^ level of 2.16 mg/dL (0.89 mmol/L), as compared to a normal reference range for Mg^2+^ of 1.82–2.32 mg/dL (0.75–0.96 mmol/L) from the U.S. National Health and Nutrition Examination Survey I study (Lowenstein and Stanton, 1986). In another study of 15 untreated hypertensive individuals given 600 mg/day of Mg^2+^ in the form of Mg^2+^ oxide, blood pressure decreased, but no difference in aldosterone levels was observed (Sanjuliani et al., 1996). In a third study that provided 600 mg of Mg^2+^ daily as Mg^2+^ oxide to 17 subjects, Mg^2+^ decreased blood pressure but again failed to significantly reduce plasma aldosterone (Haga, 1992). In this study, baseline serum Mg^2+^ levels were 1.88–1.91 mg/dL (0.77–0.79 mmol/L). In these three studies, mean baseline aldosterone levels ranged from roughly 9–13 ng/dL (240–260 pmol/L). This is on the lower side of the reference range of 5–30 ng/dL (140–830 pmol/L) determined in healthy adults on an unrestricted Na^+^ diet (Al-Dujaili and Edwards, 1978). Thus, although Mg^2+^ supplementation did not reduce aldosterone levels, these findings may be influenced by the observation that study participants exhibited neither Mg^2+^-depletion nor elevated aldosterone (e.g., from extracellular fluid volume depletion) at baseline.

Few studies have examined the response of aldosterone to oral Mg^2+^ supplementation in humans in the context of an aldosterone secreting stimulus, such as extracellular fluid volume depletion or a dietary K^+^ challenge. An exception is a study that measured aldosterone changes in response to an hour of exercise in nine men (Golf et al., 1984). Exercise increased plasma aldosterone from 9.4 ± 5.0 ng/dL (260 ± 140 pmol/L) to 19.1 ± 13.7 ng/dL (530 ± 370 pmol/L), perhaps secondary to either extracellular fluid volume depletion or increased plasma K^+^. Two weeks of daily oral supplementation with 360 mg (15 mmol) Mg^2+^ as Mg^2+^ aspartate abrogated this increase in aldosterone, leading to aldosterone levels before and after exercise that were 13.7 ± 3.2 (380 ± 90 pmol/L) and 11.9 ± 7.9 ng/dL (330 ± 220 pmol/L), respectively. Whether oral Mg^2+^ supplementation influences aldosterone secretion in response to thiazide diuretics used for hypertension, which both deplete Mg^2+^ and stimulate aldosterone secretion through fluid volume contraction, seems likely, though unreported.

What are the mechanisms by which Mg^2+^ may modulate aldosterone secretion? This effect could be mediated by a direct influence upon aldosterone-secreting zona glomerulosa cells or via modulation of upstream components the renin-angiotensin-aldosterone system. A direct effect upon adrenal function is suggested by studies showing that Mg^2+^ exerted a voltage-dependent blockade of inwardly rectifying K^+^ and Ca^2+^ channels in adrenal glomerulosa cells (Vassilev et al., 1992; Lotshaw and Li, 1996). Activity of each of these channel types modulates aldosterone secretion. Furthermore, in cultured adrenal cells, higher extracellular Mg^2+^ reduced basal aldosterone secretion (Antonipillai et al., 1997). Extracellular Mg^2+^ also attenuates stimulation of aldosterone secretion by angiotensin II. In adrenal cells in culture, increased extracellular Mg^2+^ reduced angiotensin II-stimulated aldosterone release (Atarashi et al., 1989; Antonipillai et al., 1997). This effect was also observed *in vivo*, as rats given an infusion of angiotensin II in combination with Mg^2+^ sulfate exhibited diminished plasma aldosterone, as compared with rats given angiotensin II alone (Atarashi et al., 1990). Evidence for an influence of Mg^2+^ on angiotensin II-mediated aldosterone secretion in humans is provided by a study showing that 3 weeks on a very low (<1 mEq/day) Mg^2+^ diet augmented angiotensin II-stimulated aldosterone secretion (Rude et al., 1989). This increase in aldosterone secretion was partially rescued by acute intravenous Mg^2+^ repletion with Mg^2+^ sulfate. Together, these studies suggest that extracellular Mg^2+^ decreases sensitivity of adrenal glomerulosa cells to angiotensin II-stimulated aldosterone secretion.

Studies examining the influence of Mg^2+^ on upstream components of the renin-angiotensin-aldosterone system are more mixed. In laboratory rats, 14 weeks on a Mg^2+^-deficient diet resulted in no difference in angiotensin II levels (Jin et al., 2013). However, another study examining rats on a Mg^2+^-deficient diet for 6 days found increased angiotensin II, as well as increased plasma renin activity (Sapna et al., 2006). A third study examining dietary Mg^2+^-restriction in rats found increased plasma renin activity at 2 weeks but not at 21 weeks (Laurant et al., 1999). In dogs given a low Mg^2+^ diet, plasma renin activity did not increase at any of several time-points through 90 days, although plasma aldosterone excretion did increase (Helber et al., 1972). In humans, serum Mg^2+^ was found to correlate directly with renin activity (Lind et al., 1989). In contrast, acute Mg^2+^ sulfate infusion increased plasma renin activity (Ichihara et al., 1993).

Taken together, these studies suggest that systemic Mg^2+^ depletion promotes aldosterone secretion without necessarily stimulating increased renin or angiotensin II levels. Whether this increase in aldosterone is reversible or leads to persistent aldosterone secretion (e.g., by promoting adrenal hyperplasia) remains unexplored. Although mechanisms discussed above suggest that systemic Mg^2+^ depletion should promote tubular reabsorption of Na^+^, modulation of extracellular fluid volume by Mg^2+^ has not been described. In a study examining the impact of dietary Mg^2+^ depletion upon blood pressure and fluid volume in mice, although a Mg^2+^-deficient diet increased blood pressure, it did not increase body fluid content, as measured using quantitative magnetic resonance (Pitzer Mutchler et al., 2023). However, Na^+^ overload promotes hypertension via mechanisms that may be Mg^2+^-sensitive, discussed below.

### Pro-hypertensive inflammatory processes

Na^+^ can increase blood pressure via at least two pro-inflammatory mechanisms (Kirabo, 2017). First, high Na^+^ diet increases oxidative stress in antigen-presenting cells (APCs). Peroxidation of arachidonic acid forms isolevuglandins (IsoLGs), γ ketoaldehydes capable of covalently modifying endogenous proteins. Modified proteins are presented at the APC surface, stimulating inflammation. Genetic prevention of IsoLG formation or pharmacologic scavenging of IsoLGs prevents salt-induced hypertension in mouse models (Kirabo et al., 2014; Barbaro et al., 2017). Second, high Na^+^ diet increases expression of NLRP3 (NOD-, LRR- and pyrin domain-containing protein 3), a key component of the inflammasome, in the renal medulla and other tissues (Zhu et al., 2016). The NLRP3 inflammasome catalyzes the production and secretion of the proinflammatory cytokines, IL-1β and IL-18 (Elijovich et al., 2021). Genetic or pharmacologic impairment of the NLRP3 inflammasome prevented blood pressure increases in mouse models of hypertension (Wang et al., 2014; Pitzer et al., 2022).

A Mg^2+^-deficient diet activates these hypertension-promoting inflammatory processes. In laboratory animals, dietary Mg^2+^ deficiency stimulates leukocytosis and circulating inflammatory cytokine levels (Weglicki et al., 1992; Malpuech-Brugère et al., 2000; Van Orden et al., 2006). Oral Mg^2+^ supplementation in humans suppresses circulating C-reactive protein (Mazidi et al., 2018). In mice experiencing hypertension in response to dietary Mg^2+^ restriction, circulating IL-1β levels increase (Pitzer Mutchler et al., 2023). NLRP3 and IsoLG positivity in splenic and renal dendritic cells increase to levels comparable to a high Na^+^ diet. Whether hypertension induced by dietary Mg^2+^ depletion is dependent upon activation of the NLRP3 inflammasome or production of IsoLGs was not examined, but these findings are consistent with a contribution of Mg^2+^ depletion to hypertension-promoting inflammation. Interesting questions remain regarding the mechanisms by which dietary Mg^2+^ restriction induces inflammation and whether dietary Mg^2+^ supplementation protects against high salt diet-mediated inflammation and hypertension.

## Clinical implications

The likely contributions of Mg^2+^-deficiency to increased blood pressure and to other aspects of cardiovascular disease suggest that optimal management of hypertension should include attention to Mg^2+^ balance. Clinicians should have a high index of suspicion for Mg^2+^-depletion in hypertensive patients, given that 1) dietary Mg^2+^ deficiency is common (USDA Agricultural Research Service, 2019), 2) hypertension is associated with Mg^2+^ depletion, even in untreated patients (Rodríguez-Moran and Guerrero-Romero, 2014; Rodríguez-Ramírez et al., 2015), 3) common comorbidities (such as diabetes mellitus) are also associated with Mg^2+^ depletion (Ray et al., 2023), and 4) commonly prescribed medications promote Mg^2+^ deficiency, including thiazide-type and loop diuretics, and proton pump inhibitors (Ray et al., 2023). Clinicians should not rely solely upon measurement of plasma Mg^2+^ levels for determination of Mg^2+^ depletion, since 1) less than 1% of bodily Mg^2+^ resides in the plasma, so that plasma Mg^2+^ levels do not faithfully reflect bodily Mg^2+^ stores, and 2) “normal” reference ranges typically used for plasma Mg^2+^ are likely inappropriately low (Costello et al., 2016; Micke et al., 2021). Clinicians should consider prescription of a well-absorbed oral Mg^2+^ supplement. Well-absorbed supplements include most organic salts and possibly the chloride salt, as discussed elsewhere (Ray et al., 2023). Clinicians should have a low threshold for prescribing agents that oppose urinary Mg^2+^-wasting, such as K^+^ and Mg^2+^-sparing diuretics (e.g., spironolactone or amiloride), and SGLT2 inhibitors (Ray et al., 2020). It is likely that improved Mg^2+^ balance associated with these agents contributes to improved cardiovascular benefits associated with their use (Ray, 2020).

## Conclusion

Mg^2+^ depletion likely promotes increased blood pressure via numerous mechanisms, described above, including effects on the sympathetic nervous system, vascular tone, the RAAS system, systemic Na^+^ and K^+^ balance, and inflammatory processes. Given the widespread prevalence of Mg^2+^ depletion and the tendency of some approaches to treating hypertension to induce Mg^2+^-depletion, attention to systemic Mg^2+^ deficiency has the potential to improve clinical management of hypertension and cardiovascular outcomes.
